# Intrathecal Analgesia and Palliative Care: A Case Study

**DOI:** 10.4103/0973-1075.63134

**Published:** 2010

**Authors:** Naveen S Salins, Gregory B Crawford

**Affiliations:** Palliative Medicine Consultant, Shiridi SaiBaba Cancer Hospital and Research Centre, Kasturba Medical College, Manipal University, Manipal - 576 104, India; 1 Mary Potter Senior Lecturer in Palliative Medicine, University of Adelaide, C/- Calvary Hospital, 89 Strangways Terrace, North Adelaide, 5006 South Australia, Australia

**Keywords:** Early complications, Intrathecal analgesia, Quality of life

## Abstract

Intrathecal analgesia is an interventional form of pain relief with definite advantages and multiple complications. Administration of intrathecal analgesia needs a good resource setting and expertise. Early complications of intrathecal analgesia can be very distressing and managing these complications will need a high degree of knowledge, technical expertise and level of experience. Pain control alone cannot be the marker of quality in palliative care. A holistic approach may need to be employed that is more person and family oriented.

## INTRODUCTION

The specialty of palliative medicine has emerged as a field of expertise in management of symptoms, complications, communication, decision making and psychosocial care for the patients and their families.[1] Palliative care embraces a number of different frame works and approaches to meet the needs of the “whole” person. Palliative care draws heavily on a broad spectrum of disciplines, knowledge, skills, experience and thought.[2] Though we acknowledge that palliative care focuses on a holistic relief of suffering, its main emphasis continues to be control of pain and physical symptoms. We fail to see a person beyond their symptoms and too much of suffering is left undiagnosed and unrelieved.[3]

The major symptom burden in palliative care is pain. The guidelines established by the World Health Organization (1986) regarding the basic principles of using analgesic drugs, control 90% of cancer related pain syndromes. However, 10% of patients with unrelieved cancer pain, who failed systemic treatment, still represent a significant burden of unmet need. Subcutaneous and intravenous routes may help those who have failed oral therapy and alternatively opioid rotation may improve the analgesic adverse effect balance. When all these methods are exhausted more invasive techniques such as intrathecal drug delivery system may need to be explored.[4] Less than 2% of patients with cancer pain are candidates for intrathecal analgesia.[5]

Intrathecal analgesia has definite advantages with fewer systemic side-effects and has better analgesic effects. However, the early and long term complications with intrathecal catheters are significant. Intrathecal opioids provide both spinal and supraspinal analgesia and when combined with local anesthetics, help to improve this analgesic effect. Despite the many successes and benefits of intrathecal analgesia, the potential complications that may arise can be disastrous.[6]

The WHO definition of palliative care states - “Palliative care is an approach that improves the quality of life of patients and their families facing the problems associated with life-threatening illness, through the prevention and relief of suffering by means of early identification and impeccable assessment and treatment of other problems, physical, psychosocial and spiritual.” There is an overt need to rediscover the first principles of palliative care as we tread through the focused pain and symptom control approach.[7]

## CASE REPORT

An 82-year-old man who lived independently in a retirement village was diagnosed with locally invasive transitional cell carcinoma of the bladder. He had two transurethral resections of his bladder tumor and one treatment of local palliative radiotherapy. He had long-standing excruciating pain over his penis and groin associated with recurrent urinary tract infections. He was a fiercely independent man who was cognitively sound and enjoyed gardening and driving. His wife had died several years earlier and he had a close female companion who was his major support.

He had neuropathic pain secondary to the infiltration of the pelvic neural plexus by the invasive transitional cell carcinoma. Morphine was poorly tolerated both orally and subcutaneously due to intractable nausea and vomiting. He also had borderline renal function. He had been prescribed various neuropathic regimes with minimal response. His pain remained uncontrolled on transdermal Fentanyl patch 125 mcg/hr and Gabapentin 900 mg/day. Ketamine and Lignocaine infusions did not improve pain-control.

His pain remained largely unchanged and with advice from the tertiary chronic pain management unit a decision was made to use intrathecal analgesia. An epidural catheter was initially inserted as a temporary emergency measure. As recurrent urinary tract infections were an issue, the intrathecal catheter was inserted at the tertiary hospital under appropriate antibiotic cover without an infusaport, delivering Bupivacaine and Fentanyl. The patient had an excellent response reporting total freedom from pain following the procedure.

Four days later, the dressing around his intrathecal catheter site was completely soaked with cerebro-spinal fluid (CSF). Compression bandages were applied at various sites combined with a large volume intrathecal infusion to prevent CSF leak. Two unsuccessful attempts were made to stop the flow of CSF using an epidural blood patch. The intrathecal catheter was subsequently reinserted with a subcutaneous port. For these procedures he required ambulance transfer to and from the tertiary hospital, taking 60 minutes each direction. This was very distressing for the patient causing pain and extreme fatigue. The patient developed paraparesis following an inadvertent overdose due to a malfunctioning of the intrathecal pump. Prior to this procedure he was ambulant, driving and keeping company with his female companion. He was discharged following the intrathecal procedure with partial paraparesis, an indwelling catheter, and using a wheel chair.

He was discharged to his daughter's home. Two weeks later he developed an infection at the infusaport site which required hospitalization and treatment with intravenous antibiotics. He underwent total cystectomy with ileal conduit, which led to complete resolution of his pain and removal of his intrathecal catheter. He had recurrence of his tumor six months later, with increased pain requiring reinsertion of the intrathecal catheter. He had multiple hospital admissions with pain and infections and was placed in a high level care nursing home. He died several months later.

## DISCUSSION

Intrathecal administration of analgesic agents using implantable continuous infusion system is an effective method of treating intractable pain.[8] Clinical research over the last 15 years supports the efficacy of intrathecal morphine in intractable cancer pain.[9] Multiple, other drugs have been used intrathecally with morphine for many years and hence its role is becoming increasingly more important in managing intractable pain syndromes. Complications related to the drug delivery system can occur early or late and are a source of distress to patients and family (e.g. CSF leak, pump failures, infections etc as described in this case)

Early complications of intrathecal drug delivery systems can be divided into the following categories: pharmacological, procedural, equipment, programming errors and psychological[8,10] [Table 1].

**Table 1:** Early complications of intrathecal drug delivery systems

A. Pharmacological
Nausea/Vomiting
Pruritus
Urinary hesitancy
Decreased libido, erectile dysfunction
Peripheral edema
Pharmacological paraparesis
Respiratory depression
Myoclonic jerks
B. Procedural
Infections-local or systemic
Hemorrhage/hematoma formation
Nerve damage (radiculopathy)
Occlusion or angulation (kink)
CSF leak/hygroma or spinal headache
Fibrosis
C. Equipment
Catheter dislodgement/migration
Leakage, cuts or breaks in catheter
Catheter or pump disconnection
Pump malfunctioning and pump failure
D. Programming errors
Pump mis-programming
Expertise required in handling the pump
E. Psychological
Distorted body image
Limitation of mobility and functions

Despite evidence of good pain relief, the use of spinal opioids still presents difficult technical problems.[4] The utility and cost effectiveness of intrathecal drug administration as a method to treat intractable pain is limited by the technical complications of the drug delivery systems. Pump related problems are rare with current systems, although catheter related complications remain problematic.[8] The range and incidence of complications differs widely between studies. It appears that access to technical expertise, the level of clinical experience, the care following the procedure and continuing supervision are all important factors in determining the outcome.

Catheter related complications were the most common cause of repeat procedure.[11] Catheter dislodgement was very common and its incidence varied between studies. The occurrence of migration is associated with failure to anchor the catheter to underlying fascia.[8] CSF leakage and post spinal headache are also common catheter related problems. Since the spinal catheter diameter is less than Touhy needle diameter, a CSF leak around the catheter is obvious and usually stops within several days, but occasionally persists. Fibrosis around the catheter site is common; however it is usually a late complication. This is due to a foreign body inflammatory reaction.[4] Evaluation of failures and particularly improvements in catheter design, construction, and implantation techniques can reduce complication rates and make intrathecal drug delivery systems safer and more reliable.[8]

There are studies indicating that home administering of spinal opioids is possible, safe and effective. There are significant technical difficulties in continuing care at home with intrathecal catheters. There is a need for education of the patient and family members in pump techniques, catheter care, dressing change, infection control and precautionary measures in conjunction with experienced home nurses.[8] Early pharmacological side effects from spinal opioids commonly include urinary retention and constipation. Pruritus is another common problem. Erectile dysfunction and reduced libido may be problematic and should be considered and explored sensitively. Pharmacological tolerance to spinal opioids is usually a late problem.[12]

This study reflects the spectrum of early complications of intrathecal delivery systems. This patient had an early and persistent CSF leak with post spinal headache contributing to the duration of his hospital stay. He had pharmacological paraparesis and remained wheel chair bound on discharge due to persisting lower limb weakness. He required a long term indwelling catheter due to incontinence. Although he was 82 years old he had previously lived independently. He had a female companion who was his support. Despite the real improvement in pain control, his remaining quality of life was significantly compromised. He appreciated the pain control but was disappointed about compromises needed to achieve this. The location of the access port to the intrathecal was inconvenient. He did not like having a urinary bag alongside his wheelchair. This had significantly changed his life style and future hopes. Satisfactory resolution of this man’s pain resulted in such a change in his quality of life to the extent that he felt demoralized. The hope that improved analgesia would improve his quality of life was not achieved.

The essential philosophy of palliative care is based on relieving “Total Pain” a conceptual framework having many dimensions. *Palliative* is derived from the Latin word *palliare* to cloak or shield. As palliative care clinicians we would aim to address all aspects of suffering; pain is only one of its markers. The core values of palliative care very well coincide with first principles of medical ethics i.e. autonomy, beneficence, non maleficence, and justice embracing other values such as life value, human potential, unconditional positive regard, spiritual care, cultural competence, self care and self awareness.[13,14]

Recent quality assurance data suggests that the current healthcare system provides inadequate care for dying patients.[14] The goals in end of life care may be very different from traditional medical health care. Quality indicators of good outcomes in traditional health care may not be always appropriate when evaluating end of life care. Despite evidence to suggest that pain and other symptoms can be measured and that there are processes to improve the outcomes, it is still difficult to mark these as quality indicators. Exclusive focus on pain “medicalizes” the dying process and places undue emphasis on the physical symptoms leading to the detriment of emotional, psychological and spiritual issues. Freedom from pain and other symptoms is a goal worthy of attainment however a holistic approach may need to be employed that is more person and family oriented. The conceptual framework of satisfaction in end of life care is fundamentally different from the medical care process and extends into the bereavement phase.[12]

There is strong evidence for a role for intrathecal analgesia in the management of intractable pain syndromes. However, intrathecal drug delivery systems can have significant early complications. This case demonstrates several complications. There is a need for good physical support, technical expertise and post-procedure care to achieve optimal outcomes. Pain control alone cannot be the marker of quality in palliative care. The focus needs to be individually oriented rather than having disease or symptom specific goals. A multidimensional approach between palliative care, acute care and community sectors is vital to not only achieve technical and medical goals but also to maintain the focus on patient centered informed care. This is the core philosophy of palliative care practice.
